# Diffusion MRI Findings in Encephalopathy Induced by Immunosuppressive Therapy after Liver Transplantation

**DOI:** 10.1155/2020/1015385

**Published:** 2020-02-14

**Authors:** Emanuele Tinelli, Nicoletta Locuratolo, Alberto Pierallini, Massimo Rossi, Francesco Fattapposta

**Affiliations:** ^1^Department of Human Neurosciences, “Sapienza” University of Rome, Rome, Italy; ^2^IRCSS San Raffaele Pisana, Department of Radiology, Rome, Italy; ^3^Department of General Surgery and Surgical Specialties “Paride Stefanini”, “Sapienza” University of Rome, Rome, Italy

## Abstract

Neurological complications are common after liver transplantation, as they affect up to one-third of the transplanted patients and are associated with significant morbidity. The introduction of calcineurin inhibitors, cyclosporine A and tacrolimus, in immunosuppressive regimens significantly improved the outcome of solid-organ transplantation even though immunosuppression-associated neurotoxicity remains a significant complication, particularly occurring in about 25% of cases after liver transplantation. The immunosuppressant cyclosporine A and tacrolimus have been associated with the occurrence of major neurological complications, diffuse encephalopathy being the most common. The biochemical and pathogenetic basis of calcineurin inhibitors-induced neurotoxicity are still unclear although several mechanisms have been suggested. Early recognition of symptoms could help reduce neurotoxic event. The aim of the study was to evaluate cerebral changes through MRI, in particular with diffusion-weighted images (DWI) and apparent diffusion coefficient (ADC) maps, in two patients undergoing liver transplantation after immunosuppressive therapy. We describe two patients in which clinical pictures, presenting as a severe neurological condition, early after orthotopic liver transplantation during immunosuppression therapy, showed a different evolution in keeping with evidence of focal-multifocal lesions at DWI and ADC maps. At clinical onset, DWI showed hyperintensity of the temporo-parieto-occipital cortex with normal ADC values in the patient with following good clinical recovery and decreased values in the other one; in the latter case, MRI abnormalities were still present after ten days, until the patient's exitus. The changes in DWI with normal ADC may be linked to brain edema with a predominant vasogenic component and therefore reversible, while the reduction in ADC is due to cytotoxic edema and linked to more severe, nonreversible, clinical picture. Brain MRI and particularly DWI and ADC maps provide not only a good and early representation of neurological complications during immunosuppressant therapy but can also provide a useful prognostic tool on clinical outcome of the patient.

## 1. Introduction

Neurological complications are common to all solid-organ transplantations (SOT), approximately occurring in one-third of patients; if not related to failure or compromise of the transplanted organ, they are frequently attributable to the immunosuppressive regimens [1, 2].

In fact, the introduction of calcineurin inhibitors (CNIs), cyclosporine A (CsA) and tacrolimus (Tac), in immunosuppressive regimens significantly improved the outcome of solid-organ transplantation, although immunosuppression-associated neurotoxicity remained a significant complication in the postoperative course.

Liver transplant recipients seem to develop neurological syndromes with higher incidence, between 9 and 42%, and earlier after the transplantation procedure than other organ transplant recipients [3].

Differences in the incidence of postoperative neurological complication are evident in patients with liver disease due to different etiologies, with over 40% of patients suffering from alcoholic hepatitis. A wide range of neurological side effects, both with tacrolimus and cyclosporine, have been reported. Less serious symptoms consist of tremor, headache, agitation, and sensorineural hearing loss [4].

More severe complications include seizures, hallucinations, *quadriplegia*, and visual disturbances. Speech disorder has been described, occurring in approximately 1% adults who had undergone liver transplantation, presenting as reversible spastic dysarthria, speech-activated myoclonus, speech apraxia, until a complete loss of speech [5], and a more severe condition with the involvement of consciousness, as akinetic mutism.

A peculiar picture, named PRES, characterized by reversible syndrome of headache, altered mental functioning, seizures, visual disturbances, and imaging study indicating leukoencephalopathy predominantly in the posterior regions of cerebral hemispheres, occurring in about 1–5% of patients, has been described [6]. However, the condition is not always reversible, nor is it restricted to posterior structures or white matter [5]. The biochemical and pathogenetic basis of CNIs-induced neurotoxicity are still unclear although several mechanisms have been suggested. Direct toxicity has been postulated, but blood CsA levels usually are within the therapeutic range in most patients.

Similarities between hypertensive encephalopathy and immunosuppression neurotoxicity leaded to suppose that hypertension could be a common risk factor in this syndrome [7].

Although CsA, more than tacrolimus, is a very lipophilic drug, it does not easily pass through the BBB. A possible hypothesis is that CNIs increase the permeability of BBB especially enhancing nitric oxide production that, associated to possibly anoxic injury, may facilitate dysfunction of the BEE. Moreover, low levels of cholesterol, expected in patients with significant liver failure, may increase the percentage of unbound drug predisposing to increased diffusion of CsA across the blood-brain barrier [8].

An alternative hypothesis is that neurotoxicity may result from mitochondrial impairment due to direct toxic action on the respiratory chain. Neurotoxic effects could also depend on immune dysregulation in CNS due to the pharmacologic effects of CNI-immunophilin complex [9].

Hypomagnesemia, associated to lower seizure threshold in a patient receiving CsA, posttransplant hyponatremia [10], prolonged surgical time, and pre-existing brain disease are also considered as risk factors.

Toxic effect, resulted from abnormal metabolism of CsA by liver *cytochrome P-450*, was also investigated. CsA neurotoxicity may be enhanced by pharmacokinetic and pharmacodynamic drug interactions [4].

Identification of patients at risk for neurological complications can help to stratify transplant recipients with potential reduction of perioperative risk.

Diffusion weighted images (DWI) are sequences used to reveal recent ischemic stroke. This sequence depends on the diffusion coefficient that measures the grade of translation of water molecules over small distances. Apparent diffusion coefficient (ADC) maps calculated by DWIs quantify the amount of motion of water molecules. The slow motion of the proton in a precocious stage of ischemic stroke leads to a high DWI signal although T2-weighted images do not show abnomalities. DWI are useful to differentiate cystic tumour and abscess. An abscess shows high DWI signals and low values in ADC maps, whereas cystic or necrotic tumor shows high ADC map values. Other DWI applications are used to detect the pathology of white matter [11, 12]. In order to differentiate between vasogenic and cytotoxic edema, we used DWI and ADC maps in encephalopathy secondary to immunosuppressive therapy after liver transplantation [13]. This information may be an important prognostic tool in predicting the recovery of these complications independent of the clinical status.

## 2. Case Presentation

We describe the occurrence of a severe neurological syndrome, identified as akinetic mutism, in two patients following successful orthotopic liver transplantation, however characterized by different clinical outcomes.

A 52-year-old woman and a 46-year-old man underwent liver transplantation for decompensated cirrhosis secondary to alcoholic liver disease. They had not had previous episode of hepatic encephalopathy except for transient mild cognitive slowing or history of neurological or psychiatric symptoms or previous neurological diseases.

We studied two patients before orthotopic liver transplantation. A complete physical examination by a neurologist with evaluation of cognitive performances and an EEG recording were a standard part of the pretransplantation assessment.

MR imaging was performed by Gyroscan NT Intera Philips 1.5 Tesla system. The MRI examination was performed with 5 mm slices thickness using T1-weighted spin echo (TR/TE = 600/20 ms), Proton Density, and T2-weighted (TR/TE = 2800/40–110 ms) Fluid Attenuated Inversion Recovery (FLAIR) (TR/TE/TI = 6000/100/2000 ms). Diffusion-weighted (TR/TE = 3500/120 ms) images were obtained in the axial plane, and an additional T2-weighted image was obtained in the coronal plane (TR/TE = 3000/110 ms). The ADC maps were calculated from diffusion images through the software supplied by the scanner. We drawn regions of interest (ROIs) for ADC maps analysis; all ROIs had a uniform shape and size (elliptical, 40 mm^2^), and were positioned in the temporal-parietal regions bilaterally where DWIt showed marked alterations. The ROIs placed on the images with a isotropic diffusion maps were transferred into the ADC maps to obtain the corresponding ADC values.

The neurological examinations performed in the pretransplantation assessment did not reveal any pathological signs, with exception of a fine postural tremor at limbs. The EEG recordings showed no specific abnormalities. Mild psychomotor slowing was evident during the cognitive test battery for the assessment of hepatic encephalopathy, without deficit in special domain except for minimal attentional deficit. None of the patients had previous episodes of hepatic encephalopathy or history of neurological or psychiatric symptoms before transplantation.

The initial postoperative recovery was uncomplicated, and the patients regained full consciousness after surgery. A triple immunosuppression therapy was started within 24 h of transplantation with CsA at a dose of 8 mg/Kg/day, mycophenolate mofetil, and prednisone. At third postoperative day, the patients began to be confused, manifesting psychomotor agitation, and then became mute. The neurologic disorder progressed rapidly, and during the 2 following days from onset, they were in a state of altered consciousness, in which they appeared intermittently alert. Even when they appeared awake, spontaneous motor and verbal responses were completely absent, and they were unresponsive even to noxious stimuli. Sleep-waking cycle was conserved. Neurological examination revealed oculogyric upward deviation of gaze. Oculocephalic (Doll's eyes) and corneal reflexes were elicitable. Mioclonic-like involuntary movements were sporadically observed, without EEG correlates. EEG recording showed a mild slowing of the background rhythm. They were normotensive. Cerebrospinal fluid analysis did not revealed abnormalities. Fungal, bacterial, and viral cultures were negative. Arterial blood pressure and biochemical parameters, including serum sodium, potassium, magnesium, phosphate, and cholesterol levels, were in the normal range. They did not experience abnormal fluctuation in serum sodium. Continuous monitoring of renal and liver functions did not reveal any signs of failure. Blood levels of cyclosporin were measured daily by high-performance liquid chromatography and were in the normal range.

**Figure 1:**
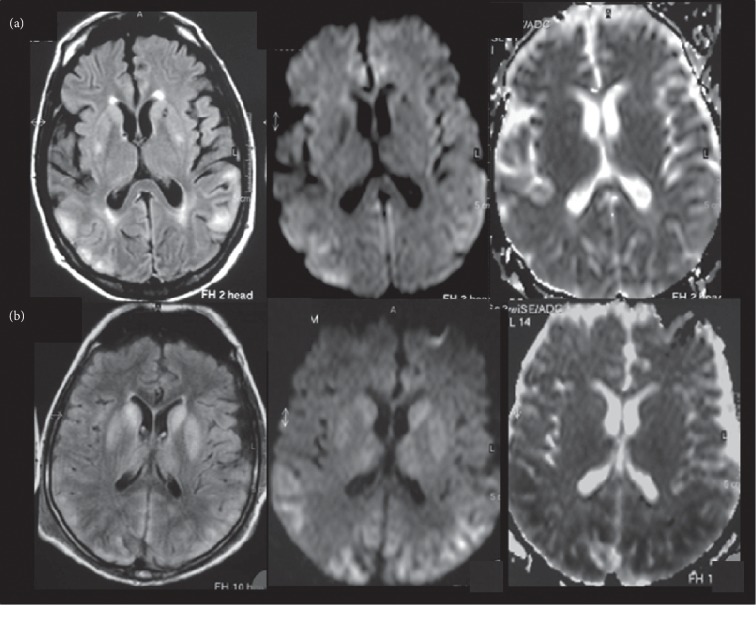
An MRI performed three days after clinical onset showed (a) T2-FLAIR and DWI bilateral and symmetrical hyperintense signal, involving the temporo-parieto-occipital cortex with normal ADC values and (b) T2-FLAIR and DWI which show the similar alterations involving the temporo-parieto-occipital cortex, with decreased ADC values; in this patient, hyperintensity in the T2-FLAIR and DWI is also evident, involving the basal ganglia and thalami without alterations on ADC maps.

An MRI performed at the time of clinical onset showed in both cases bilateral and symmetrical hyperintense signals in the T2-FLAIR and DWI, involving the temporo-parieto-occipital cortex with normal ADC values (mean ± standard deviation 0.830 ± 0.097 × 10^3^ mm^2^/sec) in one case and decreased ADC values (mean ± standard deviation 0.604 ± 0.116 × 10^3^ mm^2^/sec) in the other patient, in which was also evident hyperintensity in the T2-FLAIR and DWI, involving basal ganglia and thalami (Figure 1).

During the following two weeks, an improvement in consciousness was observed in the patient with normal ADC values. Spontaneous motor activity was evident. He was able to protrude the tongue as requested. After one month, he was able to speak and verbal and written comprehension, as well as orientation was uninjured. Only a stuttering dysarthria and dysprosody were evident. He did not show major motor and sensory deficits of the limbs, but hyper-reflexia with intermittent *clonus* at the lower limbs and bilateral Babinski sign was evident. Thus, a rehabilitation program was started.

**Figure 2:**
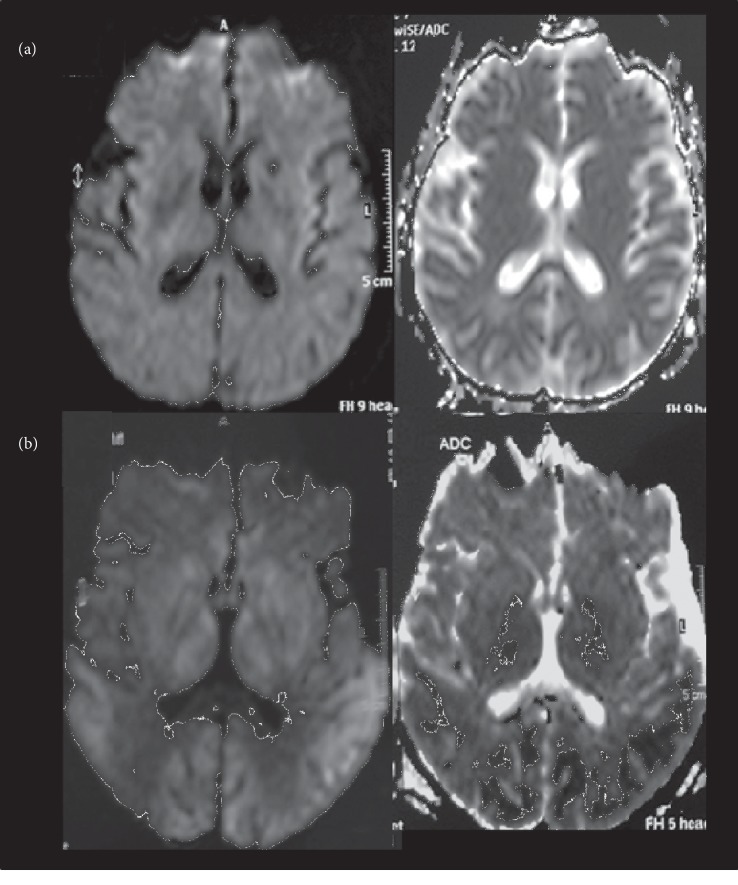
An MRI performed ten days later showed (a) DWI and ADC maps failed to show any abnormality and (b) DWI showed a persistence of bilateral and symmetrical signal abnormalities at the level of temporo-parietal-occipital cortex with reduced ADC map values.

An MRI performed ten days later revealed that the hyperintense signal was slightly decreased in the temporoparietal cortex (Figure 2(a)), with normal ADC values (mean ± standard deviation 0.894 ± 0.096 × 10^3^ mm^2^/sec); the last MRI performed two months later failed to show any abnormality either on FLAIR or DWI.

Conversely, the patient with decreased ADC values at the first MRI examination died 12 days after surgery. Neurological examination performed daily did not reveal improvement in the state of consciousness. A MRI exam performed ten days from the onset of neurological symptoms showed a persistence of bilateral and symmetrical signal abnormalities at the level of temporo-parietal-occipital cortex with reduced ADC map values (mean ± standard deviation 0.584 ± 0.121 × 10^3^ mm^2^/sec); (Figure 2(b)).

A postmortem examination showed diffuse rarefaction of the brain's white matter, swollen vascular endothelium, and perivascular macrophages.

## 3. Discussion

Together with surgical technical advances, the introduction of CNIs, CsA, and Tac, in immunosuppressive regimens significantly improved the outcome of liver transplantation. However, neurological complications occur in about 30% of liver transplant patients [4]. A wide variety of neurological adverse events can arise early or later after transplantation, suggesting the need for careful clinical assessment and follow-up, in order to promptly define the neurological syndromes. Several risk factors, such as sepsis, shock associated with multiple organ dysfunction, and graft versus host disease (GVHD) may coexist with CsA or Tac toxicity, determining the onset of encephalopathy, especially PRES; blood levels of immunosuppressive drug, however, do not correlate in most cases with the severity of neurotoxicity, suggesting that genetic differences in the CsA metabolism might be related to toxicity at therapeutic blood levels.

Clinical symptoms and neuroradiological abnormalities have been reported to mostly resolve after withdrawl of the drug [1]. However, an adverse and occasionally fatal outcome has been reported in up to 26% of the cases, and a cortical involvement of frontal regions has been reported in up to 82% of cases [13].

Normal ADC map values and high DWI signals may result from intravoxel averaging of both cytotoxic and vasogenic edema. Decreased values are caused by a prevalent cytotoxic edema. In fact, the death of the patient that was in a worse clinical status can also be explained by the neurological complications.

In conclusion, we suggest that MRI provides not only a good representation of immunosuppresant therapy complications but can also provide useful prognostic information on the patient. The hallmark of this diagnosis is the presence of vasogenic edema which is the characteristic of reversible syndrome, regardless of whether or not anterior or posterior structures are involved, and is evident at MRI as hyperintensity on both T2 FLAIR and DWI and with ADC normal or slightly high values [5, 14]. When cytotoxic edema is present or predominant, as may occur in vasospasm and ischemic complications, the ADC values are reduced which may represent an early sign of the nonreversibility of the complications. In conclusion, the diffusion-weighted sequences offer not only the possibility of diagnosing PRES but also valuable prognostic information.
